# Developments in Negative-Strand RNA Virus Reverse Genetics

**DOI:** 10.3390/microorganisms12030559

**Published:** 2024-03-11

**Authors:** Mengyi Wang, Jinyan Wu, Xiaoan Cao, Long Xu, Junhuang Wu, Haiyan Ding, Youjun Shang

**Affiliations:** 1 State Key Laboratory for Animal Disease Control and Prevention, Lanzhou Veterinary Research Institute, Chinese Academy of Agricultural Sciences, Lanzhou 730046, China; mengyiwang98@163.com (M.W.); wujinyan@caas.cn (J.W.); caoxiaoan@caas.cn (X.C.); xl645467288@163.com (L.X.); 18063447045@163.com (J.W.); haiyanding99@163.com (H.D.); 2 Gansu Province Research Center for Basic Disciplines of Pathogen Biology, Lanzhou 730046, China

**Keywords:** negative-strand RNA viruses, reverse genetics, influenza A virus, Ebola virus, peste des petits ruminants virus

## Abstract

Many epidemics are caused by negative-stranded RNA viruses, leading to serious disease outbreaks that threaten human life and health. These viruses also have a significant impact on animal husbandry, resulting in substantial economic losses and jeopardizing global food security and the sustainable livelihoods of farmers. However, the pathogenic and infection mechanism of most negative-stranded RNA viruses remain unclear. Reverse genetics systems are the most powerful tools for studying viral protein function, viral gene expression regulation, viral pathogenesis, and the generation of engineered vaccines. The reverse genetics of some negative-strand viruses have been successfully constructed, while others have not. In this review, we focus on representative viruses from the Orthomyxoviridae family (IAV), the Filoviridae family (EBOV), and the Paramyxoviridae family (PPRV) to compile and summarize the existing knowledge on reverse genetics techniques for negative-strand viruses. This will provide a theoretical foundation for developing reverse genetics techniques for some negative-strand viruses.

## 1. Introduction

Negative-stranded RNA viruses, such as the Ebola virus (EBOV), the influenza virus A (IVA), the respiratory syncytial virus (RSV), the rabies virus (RV), the measles virus (MEV), the Newcastle disease virus (NDV), the Hantaan virus (HTNV), and the peste des petits ruminants virus (PPRV), are often responsible for severe epidemics with high morbidity. Many of the epidemics caused by negative-strand RNA viruses have been listed as eradication targets by the World Organization for Animal Health (OIE) and the Food and Agriculture Organization of the United Nations (FAO) [1]. These diseases not only threaten human life and health but also affect the production of livestock and poultry. Furthermore, they cause significant economic losses and can even have adverse social impacts. Therefore, comprehensive research on negative-stranded RNA viruses is essential.

Unlike classical genetics, reverse genetics follows the path from gene to phenotype. In a broader sense, the study of patterns of inheritance and variation in living organisms reveals the relationship between an organism’s phenotype and genotype. The birth of reverse genetics is closely related to the development of genetic engineering technology. These various technologies related to reverse genetics are collectively called reverse genetics technology. Techniques such as gene knockout, gene overexpression, and RNA interference all fall under the category of reverse genetics technology. This technology allows people to manipulate genes and study the structure and function of genes, so reverse genetics has been widely used in various fields. The reverse genetics research system for various viruses that has been established using this technology, which can be achieved by creating clones of infectious viral molecules and genetically modifying them at the molecular level, is often referred to as “virus rescue”.

Reverse genetics is initially applied to DNA. Reverse genetics for DNA viruses is relatively simple, and for RNA viruses, RNA is converted to cDNA in order to study RNA at the DNA level. For positive-stranded RNA viruses, viral RNA can be synthesized by constructing an infectious full-length cDNA clone and introducing the RNA polymerase promoter. After transcription, the virus can be rescued when the transcript infects the host cell. However, the constructed full-length cDNA clone of the virus is not infectious for negative-stranded RNA. In order to make it infectious, a corresponding helper plasmid needs to be constructed. This helper plasmid can only be rescued from the infectious virus after co-transfection into cells and transcription. This process allows the virus to replicate and complete the packaging of a progeny virus. With the continuous development of negative-stranded RNA viral reverse genetics, numerous reverse genetics systems have been established for different viruses. These systems are utilized to study viral molecular characteristics, pathogenic mechanisms, and virus–host interactions, facilitating in-depth research on viral characteristics. The reverse genetic system is a powerful tool for virus research, allowing the investigation of genetic structure, function, pathogenicity, and immune mechanisms of negative-stranded RNA viruses. This will facilitate research on new vaccines and ultimately lead to the control and elimination of negative-stranded RNA viruses.

## 2. Overview of Negative-Stranded RNA Viruses

Negative-stranded RNA viruses are a significant group of pathogenic microorganisms, which include Paramyxoviridae, Rhabdoviridae, Filoviridae, Bornaviridae, Orthomyxovirdae, Peribunyaviridae, and Phenuiviridae [2]. Most of the negative-stranded RNA viral genomes are nonsegmented, while some are segmented. Among them, Paramyxoviridae, Rhabdoviridae, Filoviridae, and Bornaviridae are nonsegmented single negative-stranded RNA viruses. Orthomyxovirdae, Peribunyaviridae, and Phenuiviridae are segmented negative-stranded RNA viruses that contain 6-8, 3, and 2-8 negative-stranded RNA fragments, respectively [3].

Negative-stranded RNA viruses share common characteristics, such as being enveloped viruses, having linear and cyclic morphology, and assembling their genomic RNAs and nuclear proteins to form ribonucleoprotein complexes (RNPs). These RNPs contain viral RNA-dependent RNA polymerase (RdRp) [4]. Negative-strand RNA virus genomes do not possess the function of messenger ribonucleic acid (mRNA). When the virus genome enters the cell, it can produce complementary RNA that can serve as mRNA. The genome of a negative-stranded RNA virus and its complementary RNA molecules both bind to the viral nucleoprotein to form a nucleocapsid during viral replication. This structure prevents genomic RNA from combining with its complementary RNA to form double-stranded RNA. It ensures that there is enough positive-stranded RNA to serve as the mRNA and enough negative-stranded RNA to serve as the progeny genomic RNA. Additionally, it helps the virus evade the host immune system’s recognition of the double-stranded RNA. They rely on RdRp to complete replication of the viral genome. Generally, they replicate in the cytoplasm of the cell to produce unspliced mRNAs. However, some orthomyxoviruses and bunyaviruses transcribe and replicate in the nucleus of the cell. It is important to note that the negative-stranded RNA viral genomes are only replicated and do not participate in the process of translating viral proteins. For most negative-stranded RNA viruses that replicate in the cytoplasm, genome replication is always dependent on the synthesis of proteins. However, their mRNA synthesis is not affected by protein synthesis inhibitors, except for influenza viruses, which replicate in the nucleus [5,6,7,8].

## 3. Negative-Stranded RNA Virus Life Cycles

**Figure 1:**
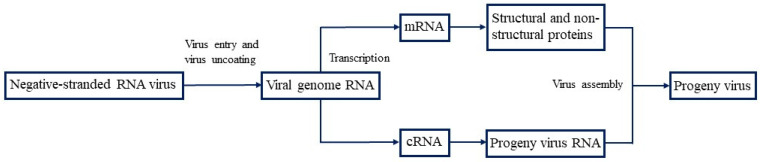
Replication cycle of a negative-stranded RNA virus. mRNA, messenger ribonucleic acid; cRNA, complementary positive-stranded RNA; progeny virus, newly synthesized virus. Negative-stranded RNA viruses infect host cells by releasing the viral genomic RNA into the cytoplasm, transcribing mRNA from the genomic RNA, and then translating the desired protein. In addition, genomic RNA copies cRNA, which serves as a template for the synthesis of viral RNA. The viral RNA is then assembled with proteins to release the newly synthesized virus.

The life cycle of negative-stranded RNA viruses includes stages such as adsorption, entry, uncoating, replication, maturation, and release. These enveloped viruses use glycoproteins on their surface for receptor binding, facilitating attachment to host cell receptors. After adsorption, the viruses enter the cell via endocytosis. The viral envelope fuses either with the cytoplasmic membrane (via the pH-independent pathway) or with the endosomal membrane of the nucleus, which has an acidic environment (via the pH-dependent pathway). Subsequently, RNPs are released into the cytoplasm to complete the uncoating [9]. Since negative-stranded RNA viruses cannot be used as translation templates, only the combination of viral genomic RNA, nucleocapsid proteins, and RdRp proteins can be used for subsequent transcription and translation. After negative-stranded RNA viruses enter the host, the viral RNA (vRNA) is transcribed by viral polymerase to produce mRNA or short leader RNA. The mRNA then serves as a template for synthesizing the proteins necessary for viral replication. During replication, the vRNA will synthesize full-length complementary RNA (cRNA). The cRNA can be utilized as a template for synthesizing a new negative-stranded RNA from the subsequent vRNA (Figure 1) [10,11,12,13,14].

## 4. Negative-Stranded RNA Virus Rescue Strategy

Virus rescue involves the creation of infectious molecular clones of RNA viruses and artificial manipulation of viral cDNA molecules to simulate virus infection in a host, ultimately leading to the generation of progeny virus particles. When rescuing viruses, it is necessary to construct full-length cDNA clones of the genome based on the specific characteristics of the virus’s life cycle and replication mechanism. This requires the utilization of the transcription system and the necessary action elements to successfully rescue the viruses. The approach used to achieve virus rescue is called the virus rescue strategy [15].

**Figure 2:**
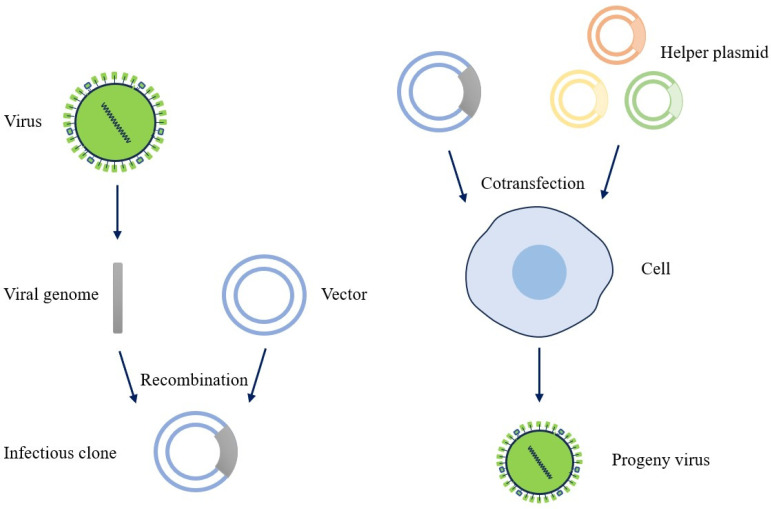
Negative-stranded RNA virus rescue strategy (as an example of a virus rescue strategy that requires helper plasmids). In the rescue of viruses, clones of infectious cDNA molecules are constructed along with helper plasmids (helper plasmids contain essential genes to ensure that full-length cDNA clones of viral genomes are infectious by activating the expression of other genes or facilitating protein synthesis), which are co-transfected into host cells. The host RNA polymerase system transcribes and synthesizes viral RNA, and the viral RNA polymerase replicates the progeny viral genome, which together with the translated viral structural proteins, assembles into a complete viral particle to achieve virus rescue.

According to the replication mode of negative-stranded RNA viral genomes, achieving in vitro rescue requires establishing an expression system for RNPs. These RNPs need to be introduced into a cell line along with the full-length genome of the virus. Subsequently, with the assistance of the host’s RNA polymerase system, the viral RNA is transcribed to produce viral RNA in vivo. This viral RNA then forms a complex with the RNPs to initiate the viral replication and assembly process, ultimately leading to the rescue of the live virus. Based on this theory, Luytjes et al. first obtained one of the RNA segments of the influenza virus through an in vitro transcription process. They then formed an active RNP by combining the purified viral nucleoprotein and polymerase and subsequently co-transfected cells with a helper influenza virus. Since the helper influenza virus not only helps the RNP to replicate and transcribe, but also provides the other seven RNA segments of the influenza virus, researchers succeeded in rescuing a chimeric influenza virus. Although this rescued virus does not represent the entire genome of the virus, it has paved the way for studying reverse genetics of negative-stranded RNA viruses and serves as a valuable reference for studying other negative-stranded RNA viruses in the future. The unsegmented negative-stranded RNA virus reverse genetic system is easy to establish. For rhabdovirus, paramyxovirus, and filovirus, the most basic and crucial point is to construct clones of infectious cDNA molecules. After constructing a full-length cDNA molecular clone of the virus genome using an exogenous expression system with high efficiency, other “key elements”, such as auxiliary plasmids, are inserted. These plasmids are co-transfected into the host cell, enabling the virus to form RNPs within the cell. With the assistance of the host’s RNA polymerase system, viral RNA is transcribed and synthesized. Ultimately, it achieves the rescue of negative-stranded RNA viruses (Figure 2). There are emerging reverse genetics systems that do not require helper plasmids, which are described in detail in a later section.

When constructing viral genomes from DNA clones, the most common method used for most RNA viruses is to clone the whole gene in segments and then ligate the segments one by one to suitable vectors. This method is considered the most efficient. After construction, rescue can be achieved through in vitro or in vivo transcription methods. In vitro transcription is a method of using RNA polymerase to synthesize RNA by using DNA as a template. This process involves the use of RNA polymerase, a DNA template containing an RNA polymerase promoter, and adenosine triphosphate to simulate the in vivo transcription process. Generally, using the T7 or SP6 transcription system, the promoter sequences of the T7 or SP6 transcription system are primed to the 5′ end of the viral genomic cDNA by PCR fusion [16]. In vivo transcription involves synthesizing RNA using the host’s RNA polymerase system after introducing the constructed cDNA into the host. The prokaryotic T7 RNA polymerase has a relatively strong transcriptional initiation efficiency and strict promoter recognition specificity. Therefore, T7 RNA polymerase-mediated virus rescue systems are widely used. And RNA polymerase in eukaryotic cells has been gradually used to establish virus rescue systems in recent years due to its convenience and the availability of mismatch repair mechanisms. The T7 RNA polymerase promoter-based in vivo rescue system involves the construction of a cDNA recombinant plasmid containing T7 RNA polymerase, the construction of a cell line that stably expresses T7 RNA polymerase, and the introduction of the recombinant plasmid is introduced into the cells, allowing for the expression of viral RNA in large quantities through the action of T7 RNA polymerase. The eukaryotic polymerase-based in vivo rescue system utilizes RNA polymerase to synthesize viruses. RNA polymerase I is responsible for transcription, producing negative-stranded viral RNA, while RNA polymerase II is responsible for synthesizing positive-stranded mRNA.

Different viruses need to develop distinct rescue strategies based on their different life cycles and replication characteristics. For example, the Orthomyxoviridae family employs several rescue strategies, including (i) reconstruction of the RNP transfection system [17,18,19], (ii) a rescue system based on the T7 RNA polymerase promoter [20,21], and (iii) a rescue system based on the RNA polymerase I promoter [22,23]. The Paramyxoviridae family employs several rescue strategies, including (i) a microgenomic system [24,25], (ii) a T7 RNA polymerase promoter rescue system [26,27], and (iii) an RNA-based polymerase II promoter rescue system [28]. The Rhabdoviridae family employs several rescue strategies, including (i) a rescue system based on T7 RNA polymerase promoter [29] and (ii) an RNA-based polymerase II promoter rescue system [30]. The Filoviridae family employs several rescue strategies, including (i) a microgenomic system and (ii) an infectious cloning system (Table 1) [31,32]. Some studies have shown that the in vivo transcription method is superior to the in vitro transcription method. In a study by Commandeur et al., while trying to rescue a cauliflower mosaic virus (CaMV), the viral cDNA was generated in vivo with a redundant nucleotide at the 5′ end of the transcript, which still successfully rescued the virus. However, when the transcript was generated in vitro using the same redundant nucleotide, the CaMV could not be successfully rescued [33].

In addition to the strategies mentioned above for rescuing negative-stranded RNA viruses, there are several other factors to consider. Scientists have discovered that when generating biologically active viral RNAs through cloning full-length cDNAs for in vitro transcription, it is crucial to synthesize a transcript with the correct 3′ end. Heterologous sequences in this region could significantly decrease infectivity [34]. The 3′ end poly(A) tail of the transcript is crucial for maintaining the infectivity of the transcript and is even necessary for the infectivity of some viruses. Not only is the 3′ end crucial for this purpose, but the presence of redundant sequences at the 5′ end can also reduce or lose infectivity of the transcript [35]. This may be attributed to the function of non-viral redundant nucleotides at the 5′ end. But for animal viruses, the infectivity of their transcripts is less affected by redundant sequences. When the nucleotides redundant at the 5′ end of the transcripts are similar lengths but differ in sequence, then the transcripts differ in infectivity [36]. In addition, the cap structure (m7GpppG) and poly(C) sequence also have a certain influence on the infectivity of transcripts. For certain viruses, the presence of a poly (C) segment at the 5′ end of their transcript can impede the synthesis of the complete virus’s cDNA or impact other characteristics of the virus. The cap structure may or may not be a critical component for some viral transcripts to ensure their infectivity [37].

## 5. Advances in Reverse Genetics of Negative-Stranded RNA Viruses

Fraser et al. were the first to utilize reverse genetics techniques to rescue an infectious T2 phage in 1957 [38]. Reverse genetic technology was first applied to DNA viruses by Goff et al. in 1976. They established the first DNA viral reverse genetics operating system and achieved the in vitro “rescue” of SV40 by transfecting SV40 DNA with artificial mutations [39]. The replication cycle of RNA viruses, except Retroviruses, does not involve a DNA stage. The RNA transcribed from the full-length cDNA of their genomes is not infectious by itself. Instead, it must form an RNP with nuclear coat protein, RdRp, and other components in order to carry out replication and packaging of viral particles. As a result, research on reverse genetic manipulation of RNA viruses lags behind that of DNA viruses. However, with advances in molecular biology, Taniguchi et al. successfully rescued the positive-sense RNA virus-phage Qbeta in 1978 [40]. Subsequently, Racaniello et al. cloned an infectious poliovirus in 1981 [41]. The success of these two studies laid the foundation for reverse genetics of RNA viruses. However, negative-stranded RNA viruses differ from other RNA viruses in that neither the genome of negative-stranded RNA viruses nor their cDNAs are infectious. As a result, the manipulation of negative-stranded RNA viruses using reverse genetics is much more complex and challenging. In addition to obtaining precise full-length clones of the cDNAs, it is necessary to conjugate them with nucleocapsid proteins and RNA polymerase proteins. Furthermore, co-transfection of the appropriate cells is required to obtain infectious viral particles. Therefore, the number of successful examples of rescuing negative-stranded viruses has been much lower than that of positive-stranded viruses. A chimeric influenza virus was first rescued through in vitro transcription in 1989. This breakthrough pioneered the study of negative-stranded RNA reverse genetics and served as a guiding reference for rescuing other negative-stranded RNA viruses. In this paper, we illustrate the progress of reverse genetics in negative-stranded RNA viruses by selecting one virus from each of the Orthomyxoviridae family (IVA), the Filoviridae family (EBOV), and the Paramyxoviridae family (PPRV).

### 5.1. Advances in Reverse Genetics of IVA

**Figure 3:**
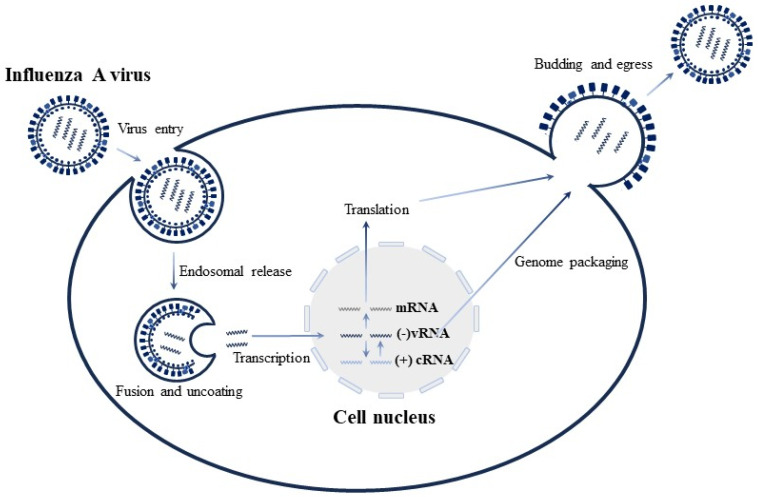
Replication cycle of IVA. IVA, influenza virus A; (−)vRNA, viral negative-stranded RNA; (+)cRNA, complementary positive-stranded RNA. When an influenza virus infects the recipient cell, the virus recognizes the receptor on the surface of the recipient cell, enters the cell through membrane fusion, and the vRNPs in the virus are released into the cytoplasm, and then the vRNPs are transported to the nucleus for viral genome replication and transcription. RNA is transcribed into mRNA and cDNA. The mRNA is then translated into viral protein, while the cDNA is utilized as a template for synthesizing the negative-strand RNA of the progeny virus.

Influenza viruses can be categorized into three types: A, B, and C. These viruses are classified into subtypes based on the antigenicity of the hemagglutinin (HA) and neuraminidase (NA) on their outer membranes. There are 18 known HA subtypes and 11 NA subtypes, and HA and NA can be freely combined resulting in more than 100 combinations. For example, the first strains of influenza virus isolated from humans are H1N1 and H7N9, which have had a significant impact on society. IVA is an acute respiratory infectious disease caused by influenza viruses that can infect humans, other mammals, and birds. It leads to extremely high mortality and morbidity worldwide and is highly prone to mutation, resulting in influenza pandemics that pose significant harm to humans and animals [42]. IVA belongs to the Orthomyxoviridae family and has a genome length of approximately 12,000–15,000 nucleotides. The genome is divided into eight segments that encode the production of more than 12 functional proteins [43]. The viral RNA (vRNA) contains a central coding region with non-coding regions at either end. These non-coding regions act as promoters to initiate genome replication and gene transcription through the viral polymerase complex. Its genetic material, single-stranded, negative-sense RNA, binds to the nucleoprotein (NP) and then associates with the RNA polymerase subunits (PB1, PB2, and PA) to form the RNP. Unlike most other RNA viruses, IVA replicates in the nucleus of infected cells, and their vRNAs and mRNAs are translocated to the cytoplasm during the early and late stages of infection (Figure 3). However, transcripts, which serve as templates for RNA synthesis in viral particles, are not translocated to the cytoplasm during viral infection [44]. In addition, the presence of all RNA polymerase bodies and NPs is necessary for viral transcription and replication [45,46]. This requires the simultaneous presence of eight functional RNPs in the nucleus, which is the cellular compartment where vRNAs need to be synthesized or delivered. However, this combination of RNPs poses a significant challenge to the development of the Influenza virus reverse genetics system [47,48].

**Figure 4:**
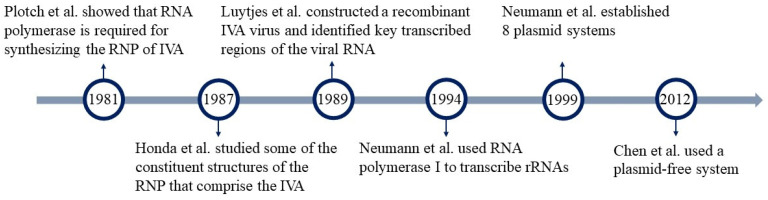
Advances in reverse genetics of IVA. IVA, influenza virus A [51,52,53,54,55,56].

IVA replication needs three polymerase subunits, PB1, PB2, and PA, and the nuclear protein NP. Therefore, to construct a reverse genetic system for the IVA, a total of eight viral RNA and four protein components are required. These components can be successfully constructed in the appropriate proportion [49,50]. Plotch et al. and Honda et al. found that the RNP complex responsible for initiating IVA replication consists of vRNA, RNA polymerase, and NP protein [51,52]. In 1989, Luytjes et al. replaced the NS gene of IVA with a recombinant RNA of chloramphenicol acetyltransferase (CAT) and combined it with extracted RNP in vitro to form RNPs. Using purified IVA polymerase protein and helper viruses, the recombinant RNA was amplified, expressed, and packaged into viral particles. This resulted in RNA containing CAT and offspring viruses with eight other fragments. Additionally, it has been discovered that the 26nt at the 5′ end and the 22nt at the 3′ end of influenza virus RNA are crucial regions for vRNA transcription and replication [53]. Seong et al. subsequently rescued the virus by reconstructing the RNP after treating it with microsphere nuclease. Neumann et al. (1994) utilized RNA polymerase I to transcribe rRNA, which can be recognized by the influenza RNA polymerase and subsequently assembled into infectious viral particles. This method does not require protein purification or in vitro RNA transcription, making it more convenient compared to the method proposed by Luytjes et al. [54]. Subsequently, Neumann et al. established a new reverse genetic operating system in human embryonic kidney cells (293T) by transfecting eight plasmids for IVA in 1999. This system no longer requires the involvement of auxiliary viruses. This IVA was completely generated from cloned cDNAs, creating a new system that operates solely on plasmids. This approach offers increased efficiency and convenience [55]. A plasmid-free system has also emerged, which can be synthesized directly from sequencing known viruses to produce either eight naked DNA sequences or double-stranded naked DNA. Translation of DNA can be initiated bi-directionally by adding a promoter, such as polⅠ-tⅠ, and the cmv-bgh polyA signal to both ends of each gene. Based on this concept, Chen et al. utilized fusion PCR to connect influenza virus genes to the polⅠ-tⅠ element and successfully rescued influenza viruses. They showed partial dependence on plasmids (retaining the PB2, PBI, PA, NP, and NS plasmids) or no dependence on plasmids at all [56]. Subsequently, Krumbholz et al. used synthetic HA and NA to rapidly rescue vaccine candidates, such as H7N9 [57]. Dormitzer et al. utilized viral RNA expression constructs encoding HA and NA, along with plasmid DNA encoding the viral backbone genes, to rescue the virus through transfection into cells, which increased the yield of vaccine antigen HA [58]. The plasmid-free rescue system is expected to accelerate the stockpiling of influenza vaccine candidates for a rapid response to pandemic influenza outbreaks. Furthermore, it completely avoids the problem of drug resistance caused by plasmid residues. The rescued influenza viruses do not need to be decontaminated by plasmids, making it more suitable for vaccine production processes (Figure 4).

### 5.2. Advances in Reverse Genetics of EBOV

EBOV belongs to the Filoviridae family and is a highly infectious virus that can cause the severe and often fatal disease known as Ebola virus disease. This disease affects humans and other primates, causing symptoms such as fever, nausea, vomiting, diarrhea, internal and external bleeding, and multiple organ dysfunction syndrome. It is highly contagious and has a high morbidity rate [59,60]. EBOV has a total of 18,959 bases, making it the longest among the single-stranded, negative-stranded RNA viruses. The virus has an envelope, and its particles are generally filamentous and sometimes segmented [61]. The gene encodes seven structural proteins, namely nuclear protein (NP), polymerase cofactor (VP35), matrix protein (VP40), glycoprotein (GP), protein VP30, matrix protein (VP24), RNA-dependent RNA polymerase (L), and a non-structural small glycoprotein sGP. Viral RNA is spirally wound and complexed with NP, VP35, VP30, and L proteins, and the helical nuclear capsid is surrounded by an outer envelope [62,63]. There are five subtypes of EBOV, including EBO-Zaire, EBO-Sudan, EBO-R, EBO-CI, and the Tai Forest virus. EBO-Zaire and EBO-Sudan have high pathogenicity and mortality rates for humans [64,65].

**Figure 5:**
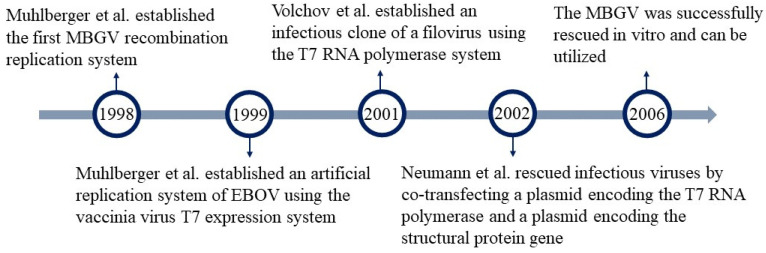
Advances in reverse genetics of EBOV and MBGV. EBOV, Ebola virus; MBGV, Marburg virus [66,67,69,70].

The Filoviridae includes only the EBOV and the Marburg virus (MBGV), which are very similar in terms of their morphology, genetic structure, protein composition, and replication and transcription processes. The research progress of reverse genetic operations for both viruses is closely related, so they will be described together. Muhlberger et al. (1998) first demonstrated that three of the four nucleocapsid proteins of MBGV, NP, VP35, and L are sufficient to mediate replication and transcription of the MBGV-specific monocistronic genome. This study established the first MBGV recombinant replication system. The system involved the MBGV microgenome, which contained the leading and trailing regions of the MBGV genome, as well as the CAT gene [66]. In 1999, an artificial replication system for EBOV was developed using the cowpox virus T7 expression system. This system demonstrated specific transcription and replication of an artificial single cis-replicon mini-replicon through the expression of reporter genes and the detection of transcribed and replicated RNA species. It was found that EBOV-specific transcription relied on the presence of a fourth nuclear capsid protein, VP30 [67]. However, the T7 RNA polymerase system’s promoter could not be recognized by the RNA polymerase of eukaryotic cells. Therefore, Groseth et al. replaced the T7 promoter in the above system with a promoter that can be recognized by RNA polymerase I in 2005. This system is more efficient. [68]. Volchov et al. established an infectious clone of filovirus in 2001. By utilizing the T7 RNA polymerase system, they were able to recover the infectious virus from the cloned cDNA. This allows for genetic manipulation of the virus, enabling the study of the mechanisms behind the high pathogenicity of the EBOV [69]. In 2002, Neumann et al. also rescued infectious viruses by co-transfecting plasmids encoding T7 RNA polymerase and structural protein genes [70]. Subsequently, the MBGV was successfully rescued in vitro, and the rescued virus showed almost no differences in virus particle morphology, infectivity, and growth kinetics compared to wild-type strains, making it suitable for application [71,72]. This represents the maturation of an infectious clonal rescue system for filoviruses. Later, Tianyu Gan et al. established a new reverse genetic system for EBOV that only requires a single viral RNA genome to be transfected into a cell line expressing the viral NP, VP35, VP30, and L proteins. This modification enables efficient replication of EBOV (Figure 5) [73].

### 5.3. Advances in Reverse Genetics of PPRV

Peste des petits ruminants (PPR) is an acute viral infectious disease caused by the PPRV. PPRV is one of the most significant viral pathogens affecting livestock, particularly small ruminants such as sheep, goats, and deer. The PPRV belongs to the Paramyxoviridae family. Its genome consists of nonsegmented, single-stranded, negative-sense RNA surrounded by nucleoprotein N (N). This nucleoprotein N forms a helical nucleocapsid and combines with RNA-dependent RNA polymerase and cofactor phosphoproteins to form an rRNP. This complex is the smallest functional unit of replication for negative-stranded viruses. The length of the PPRV genome is 15,948 nucleotides (a variant with an additional 6 nucleotides has been detected in recent epidemics in China). This length conforms to the paramyxovirus “six-base rule” and allows the genome to replicate normally with the addition or removal of one or two nucleotides to its micro-mutated genome, in contrast to other viruses that strictly adhere to the “six-base rule” [74]. PPRV contains six transcriptional units, namely N, phosphoprotein (P), matrix protein (M), fusion protein (F), hemagglutinin protein (H), and large protein (L). Additionally, two other non-structural proteins, C and V, are generated from the P open reading frame. This was achieved by utilizing alternative start codons and RNA editing, respectively, in the order 3′-N-P-M-F-H-L-5′ [75]. There is only one serotype of PPRV, and its strains are divided into four different lineages. Lineages I-II are spread in West Africa, Lineage III is found in East Africa, and Lineage IV is primarily found in the Middle East and Asia [76].

**Figure 6:**
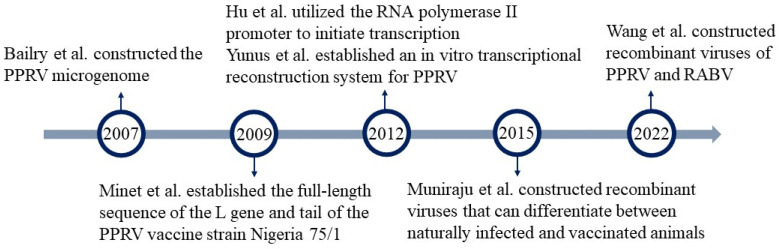
Advances in reverse genetics of PPRV. PPRV, peste des petits ruminants virus [74,78,80,81,82].

The reverse genetic system of the PPRV is later compared to other viruses of the same genus. Bailey et al. first attempted to construct a PPRV microgenome in 2007 and studied its expression in transfected cells. They found that the required elements for rescuing the PPRV microgenome were N, P, and L proteins, antisense PPRV cDNA, PPRV genome promoter (GP), and PPRV anti genome promoter (AGP), with hepatitis D virus ribozymes (HDVRZ) on both sides. The efficiency of small genome rescue depends on protein–protein interactions and RNA–protein interactions, and its expression under homologous and heterologous protein and promoter combinations was studied, as well as its compliance with the “six base rule” in vitro [77]. Minet et al. (2009) established the full-length sequence of the L gene and tail of the PPRV vaccine strain Nigeria 75/1. This study marked the first time that reverse genetics was employed to determine the N, P, and L proteins, as well as the leading and trailing sequences of PPRV Nigeria 75/1 for microgenomic expression [78]. In 2012 Yunus et al. first established an in vitro transcriptional reconstitution system for PPRV using RNP complexes purified from infected cells and recombinant L–P complexes expressed in insect cells. Both complexes are capable of synthesizing all mRNA species in vitro and exhibit a polarity gradient in transcription [79]. Hu et al. (2012) successfully recovered PPRV by inducing transcription of the full-length viral antigenome using the RNA polymerase II promoter. This technique can be employed to create viruses that express tracer proteins, such as green fluorescent proteins (GFPs), and maintain stable GFP expression for at least 10 generations [80]. This newly established reverse genetics system for PPRV provides a novel approach to vaccine construction by utilizing PPRV as a vector. Muniraju et al. (2015) developed a reverse genetics system for the PPRV Nigeria 75/1 vaccine strain. They successfully rescued the PPRV Nigeria 75/1 vaccine strain by inserting the GFP gene, resulting in positively labeled recombinant viruses. Additionally, they mutated the C77 locus on the H gene to obtain negatively labeled recombinant viruses. This method allows for differentiation between naturally infected and vaccinated animals [81]. Wang et al. (2022) successfully constructed recombinant viruses of PPRV and RABV by utilizing the rabies virus full-length infectious clone plasmid pD-SRV 9-PM-LASV as the backbone. They inserted the vesicle membrane glycoprotein H or F genes of PPRV into the backbone plasmid. The viruses constructed in this study have demonstrated good proliferative activity and stability, making them potential candidates for bivalent inactivated vaccines to prevent PPRV and RV in domestic animals (Figure 6) [82].

## 6. Reverse Genetics in Animal Virus Research Applications

### 6.1. Applications in the Study of the Structure and Function of Viral Genomes

With the development of reverse genetics, some technology such as gene knockout, gene overexpression, or construction of chimeric viruses can be employed to study the structure or function of viral genes. Rieder et al. used the constructed FMDV infectious cDNA to study the close relationship between the poly(C) sequence and the infectivity of the virus. They constructed infectious cDNAs containing 2, 6, 16, 25, and 35 cytosine residues. They found that the transcripts of these cDNAs exhibited similar infectivity. However, the viral RNAs containing 6 to 35 cytosine residues grew faster and were able to reach viral titers comparable to those of wild viruses [83]. Using the construction of infectious clones of Equine viral arteritis (EVA), Dobbe et al. constructed chimeric viruses by replacing the EAV glycoprotein GP(5) and vesicular membrane protein (M) with genes from other RNA viruses. They then examined the transportation of the hybrid GP(5) and M proteins to the Golgi complex. Further studies revealed that the two chimeric viruses were still able to infect BHK-21 cells or RK-13 cells, but porcine reproductive and respiratory syndrome (PRRSV) and lactate dehydrogenase-elevating virus (LDV) were unable to grow on the two types of cells, suggesting that the extracellular region of the EAV GP(5) protein is not a major determinant of the histophilicity of the virus [84].

### 6.2. Applications in the Study of Viral Genome Replication and Expression Mechanisms

The use of reverse genetics technology can effectively study the replication and translation mechanisms of viral genomes. It allows for the analysis of the effects of genetic manipulations on the replication and expression levels of viral genomes. This, in turn, can be utilized to study the molecular mechanisms of their regulation. In the case of paramyxoviruses, the “six-base principle” was first discovered in the Sendai virus using reverse genetics, which is of great significance for the in vitro rescue of paramyxoviruses [85]. Furthermore, by constructing a deletion mutant of the dengue virus, Cahour et al. found that the non-coding region (NCR) of the dengue virus genome is associated with the replication and virulence of the virus. They found that bases 55 to 72 in the long stem region of the 5′ NCR secondary structure are essential for viral replication. The deletion of bases 82–87 in the 5′ NCR is associated with viral pathogenicity. Removing these bases can decrease the virus’s pathogenicity towards the host [86].

### 6.3. Applications in the Study of Novel Vaccines and Antiviral Drugs

Reverse genetics has also greatly contributed to the development of RNA vaccines. These vaccines can synthesize recombinant viral RNA containing exogenous genes through the RNA polymerase system in vitro. Subsequently, animals can be immunized with it to stimulate the immune response. The first attempt at an infectious clonal vaccine for FMDV was made by McKenna et al. They constructed FMDV missing the RGD sequence based on FMDV A12 infectious cDNA. The immunized cattle showed a good immune response [87]. Ward et al. also conducted a similar study where they constructed a genetically engineered virus missing the cellular adsorption site coding sequence. They introduced it into experimental animals, which exhibited a strong immune response and produced neutralizing antibodies to combat the strong virus attack [88].

### 6.4. Application in the Development of Novel Viral Vectors

Compared with traditional live viral vectors, RNA viruses as vectors offer advantages such as easy handling, high expression of immunogens, and a wide host spectrum. Many viruses have been used as vectors to express heterologous viral proteins and have shown significant progress. Pushko et al. successfully expressed the influenza virus HA gene or the Lassa virus N gene using a vector system based on the Eastern equine encephalitis virus [89]. After establishing an infectious clone of the Sindbis virus, Xiong et al. replaced the structural gene portion of SINV with the CAT gene. As a result, a recombinant virus was rescued which could express CAT polypeptides at high levels in host cells. This laid the foundation for the development of the Sindbis virus as a vector [90]. The weak NDV vaccine strain can also be used as a viral vector. Nakava et al. constructed a recombinant Newcastle disease virus rNDV/BI-HA expressing the HA gene of the influenza virus by inserting the HA gene of influenza virus A/WSN/33 into the genome of the weak strain of NDV between the M gene and the P gene. Mice immunized with a high titer of antibodies to HA were protected against a lethal dose of the influenza virus A/WSN/33 [91].

## 7. Discussion

Negative-stranded RNA viruses differ from positive-stranded RNA viruses in that negative-stranded RNA viruses or nucleic acids isolated from virus-infected cells are not capable of directly infecting cells. As a result, the development of a reverse genetic operating system for negative-stranded RNA viruses is lagging behind. However, in recent years, with the increasing maturity of more and more negative-stranded RNA viruses with a reverse genetic operating system, reverse genetics has become an important tool for studying the interaction between viruses and their hosts. This technology allows for the manual manipulation of viral genomes to study the function and role of genes. It also provides a foundation for the development of new recombinant viruses or vectors for vaccines and gene therapy.

In this review Orthomyxoviridae (IAV), Filoviridae (EBOV), and Paramyxoviridae (PPRV) were selected as representatives of negative-stranded RNA viruses. Among these three viruses, the reverse genetic system of the IVA has been established the earliest and is well-developed. The reverse genetic system of the EBOV has matured over time, but the reverse genetic operating system of the PPRV is still under development. The reverse genetic system of the PPRV is faced with the following problems: (i) The whole genome length of PPRV is 15,948 nucleotides, and the accurate construction of its cDNA is relatively challenging. (ii) There is a significant challenge to rescuing PPRV, and even if it can be successfully rescued, the efficiency of the rescue is low. (iii) The established PPRV reverse genetic manipulation system is not stable enough for use in research. Since both NDV and PPRV belong to the family Paramyxoviridae, the reverse genetic system of NDV is of great reference value for establishing the reverse genetic system of PPRV. The reverse genetics of NDV lays the foundation for the development of a valuable recombinant vaccine capable of expressing either its own mutant protein or an exogenous protein. This provides an opportunity to investigate its application as a recombinant vaccine, a multivalent vaccine candidate for poultry, and a vaccine vector for other animal species and humans. Peeters et al. first rescued the recombinant Newcastle disease virus from the lentogenic strain LaSota in 1999 [92]. This was followed by the development of several NDV rescue systems and advancements in NDV reverse genetics. NDV reverse genetics is becoming increasingly mature. Recently, Garcia, S. et al. described a successful technique for recovering infectious clones of NDV from full-length cDNAs. They also proposed a targeted mutagenesis scheme to attenuate fusion protein cleavage sites, as well as a gene substitution approach for fusion and hemagglutinin-neuraminidase. This study has been highly informative in establishing a reverse genetic operating system for PPRV [93]. In addition, some of the more mature viruses studied in reverse genetics can be utilized by inserting the genes of the PPRV as exogenous genes into that virus as transcription units. This allows the virus to become a vector of the PPRV [94]. Reverse genetics continues to evolve, utilizing the reverse genetic manipulation platform to investigate the mechanisms of expression regulation and pathogenesis of many viruses that pose a threat to human life. This research lays the foundation for the development of novel vaccines. Therefore, it is necessary to make good use of this technological platform to conduct in-depth research. Simultaneously, it is crucial to strengthen precautionary measures to prevent recombinant viruses from escaping the laboratory and posing a threat to biosafety.

## Figures and Tables

**Table 1:** Types of negative-strand RNA viruses and their reverse genetic system.

Virus Type	Reverse Genetics System
Orthomyxvirdae	Reconstruction of RNP transfection system Based on T7 RNA polymerase promoter rescue system Based on RNA polymerase I promoter rescue system
Paramyxoviridae	Microgenomic system Based on T7 RNA polymerase promoter rescue system Based on RNA polymerase II promoter rescue system
Rhabdoviridae	Based on T7 RNA polymerase promoter rescue system Based on RNA polymerase II promoter rescue system
Filoviridae	Microgenomic system Infectious cloning system
